# Review: Magnetic resonance imaging techniques in ophthalmology

**Published:** 2012-10-12

**Authors:** Laura Fanea, Andrew J. Fagan

**Affiliations:** 1 Department of Biomedical Physics, Physics Faculty, Babes-Bolyai University, Cluj-Napoca, Romania; 2 Department of Radiology, Cluj County Emergency Hospital, Cluj-Napoca, Romania; 3 Centre for Advanced Medical Imaging, St. James’s Hospital Dublin / University of Dublin Trinity College, Ireland

## Abstract

Imaging the eye with magnetic resonance imaging (MRI) has proved difficult due to the eye’s propensity to move involuntarily over typical imaging timescales, obscuring the fine structure in the eye due to the resulting motion artifacts. However, advances in MRI technology help to mitigate such drawbacks, enabling the acquisition of high spatiotemporal resolution images with a variety of contrast mechanisms. This review aims to classify the MRI techniques used to date in clinical and preclinical ophthalmologic studies, describing the qualitative and quantitative information that may be extracted and how this may inform on ocular pathophysiology.

## Introduction

The eye acts like a camera as it can refract light and produce a focused image, which then stimulates the neural responses that give rise to vision. According to statistics of the World Health Organization in 2002, there were 161 million people affected by visual impairment worldwide, of whom about 37 million were blind [1]. The largest proportion of blindness is related to aging, while the leading causes are cataracts. Furthermore, age-related macular degeneration is expected to increase in significance as a cause of blindness due to the growing number of people over 70 years of age [1].

Since at least 1552 BC, the earliest ophthalmologic records in the Egyptian Ebbers’ Papyrus, ocular diseases in general, and intraocular inflammation in particular, have been recognized as a threat to sight [2]. More than 3,000 years later, in 1585, 113 ocular diseases were described in an ophthalmologic treatise [3]. Despite the first modern ophthalmologic treatise being published in 1707 [4], there nevertheless remains a limited number of studies of in vivo eye disease in humans, and hence more information is required to improve the interpretation of eye images, which are routinely acquired using a variety of imaging modalities.

Color fundus photography, fluorescein angiography, indocyanine green angiography, optical coherence tomography, high-resolution ultrasound, and magnetic resonance imaging (MRI) are the ocular imaging techniques most often used clinically [5,6]. Optical imaging techniques represent the major modalities of eye investigation at present but have limitations as diagnostic and prognostic tools. Thus, while they have higher resolution compared to MRI techniques, optical imaging requires an unobstructive pathway of light from the cornea through the lens and the retina, which restricts its use in many instances. Ultrasonography, on the other hand, does not have such a deep tissue penetration when compared to MRI [5-7]. MRI techniques are useful to refine and clarify difficult diagnoses and to study disease mechanisms and the effect of therapies [6].

Most of the numerical information provided by MRI is based on measurements of pixel intensity, providing values for the signal-to-noise ratio (SNR), contrast-to-noise ratio, T_1_, T_2_ relaxation times [8], and coefficients characterizing the water diffusion [8,9] or ocular perfusion [8-10]. The information provided can be used for the differentiation of abnormal and normal tissues, the detection of tumors, and for cellular activity analyses. In functional MRI (fMRI) studies, pixel intensity information has been used to measure the response of the brain or even the retina to different stimuli [11]. Image segmentation analysis is sometimes used to extract numerical information in cluster regions with similar pixel intensity. Several segmentation techniques have been proposed for mapping T_1_ or for detection of abnormal tissues [12,13]. Measurements of distances, areas, or volumes can also be performed, and retinal thicknesses have been measured from MRI images of normal subjects and patients with ocular disease [14].

A description of the range of MRI techniques used both with and without contrast agents in animal models of disease and in humans for clinical ocular imaging is presented in this paper. The review begins with a brief overview of MRI physics and the field of ocular MRI before moving on to discuss MRI studies that either did or did not use exogenously administered contrast agents. Future directions for the field are then discussed.

## Basics of magnetic resonance imaging

MRI images represent maps of the nuclear magnetic resonance (NMR) signals emitted by magnetically labeled nuclei. To acquire an MRI image, a sample is placed in a static magnetic field which, for the majority of modern clinical systems, is generated by a solenoid made of superconducting wires. An additional transitory oscillating radio frequency (RF) field is generated by a transmitter coil placed in the sample proximity. The static and oscillating magnetic fields label specific nuclei in the sample. Specific nuclei, usually ^1^H nuclei in water molecules but also in fat molecules or macromolecules, interact with the applied magnetic fields. After switching off the additional RF field applied to the sample, the labeled nuclei in the sample will induce NMR signals in a receiver coil placed in the vicinity of the sample and tuned to the specific NMR frequency. These NMR signals are spatially encoded along the x, y, and z directions by three sets of magnetic field gradient coils. The preamplified and spatially encoded NMR signals are then processed to form an MRI image.

The amplitude of the NMR signal induced by the magnetically labeled nuclei in a region in a sample depends on the concentration of the imaged nuclei in that region and the relaxation properties of the nuclei, characterized by the T_1_, T_2_, and/or T_2_* relaxation times. The amplitude of the NMR signal emitted by the magnetically labeled nuclei in a region in a sample is directly proportional to the concentration of the nucleus imaged in that region of the sample; it decreases exponentially with time with a time constant T_2_ or T_2_*, which is the spin–spin relaxation time of the nucleus imaged. Its dependence on the spin–lattice relaxation time, T_1,_ is different for different MRI acquisition protocols [8].

Conventional MRI detects signals only from mobile hydrogen (^1^H) nuclei that have sufficiently long T_2_ relaxation times (i.e., greater than approximately 10 ms). The T_2_ of the less mobile ^1^H nuclei, which generally are associated with macromolecular structures, such as proteins, and membranes in biologic tissues, are too short (i.e., less than 1 ms) to be detected directly in conventional clinical MRI [15].

^1^H is the most abundant NMR nucleus in the body. ^1^H nuclei in liquids, including water, are mobile and have much longer T_2_ relaxation times compared to the more rigid ^1^H nuclei present in macromolecules. In the case of ^1^H nuclei characterized by sufficiently long T_2_ relaxation times, the spatial encoding gradients can be played out between excitation and acquisition of the NMR signal, before the signal has completely decayed [15]. Approximately 70% of the body comprises water, and 99.98% of naturally occurring hydrogen nuclei are the ^1^H isotope. ^1^H also has the highest NMR sensitivity of any nucleus [16], so clinical MRI usually images ^1^H nuclei in water molecules. ^1^H nuclei in macromolecular structures or in membranes can also be imaged indirectly using magnetization transfer techniques or directly at higher magnetic field strengths, using special MRI acquisition protocols. Nuclei found in low concentrations in the human body can be imaged using MRI techniques if a compound containing those nuclei can be introduced as a contrast agent in the region of the body that needs to be imaged. These MRI imaging techniques can be used for specific targeting and cellular imaging to evaluate response to therapies. Such an example is that of fluorine (^19^F) MRI. The ^19^F nuclei exist in high concentrations in the bone and teeth but in concentrations below the MRI detection limit in normal wet tissue. The lack of any background signal from most of the body provides ^19^F MRI with a potentially extremely high contrast-to-noise ratio and specificity. The ^19^F nucleus has a 100% natural abundance and resonates at a frequency that is 94% of that of ^1^H. Its NMR sensitivity is 83% of that of ^1^H, its SNR being about 89% of ^1^H per nucleus. For ^19^F MRI to produce an image quality similar to that of ^1^H MRI, the agent requires a high density of ^19^F nuclei in the molecule in addition to a high concentration of the molecule in the tissue of interest [17].

Pioneering work in physics between 1666 and 1945 made possible the first detections of NMR signals [18] in liquids and solids [19]. In 1971, the significantly different proton relaxation times of tumors and normal tissues demonstrated the clinical potential of the NMR technique [19]. During the late 1970s and the early 1980s, several groups showed promising results of MRI in vivo. Since then, MRI has rapidly developed, both in whole body clinical applications and in microscopy. Various contrast agents have been developed to enhance the NMR contrast between tissues or for in vivo cellular detection by MRI. Most advances, however, have been made in the development of new imaging protocols (pulse sequences) and MRI hardware [19].

## Basics of ocular magnetic resonance imaging

The eye is a superficial and anatomically distinct organ with structures that need to be visualized with high spatial resolution. Some of its structures have a high water content. For example, the human aqueous humor is a clear liquid having properties similar to that of water; it fills the anterior segment of the eye. The human vitreous humor is an avascular jelly containing 98% water and fills the posterior segment of the eye [20]. MRI contrast between different eye structures can, therefore, be obtained based on the differences of their water content. MRI contrast between the eye structures may also be achieved based on differences of the relaxation properties of the ocular structures, characterized by their T_1_, T_2_, and T_2_* relaxation times [8].

Contrast agents may be administered orally or by injection to provide enhanced contrast if needed [8,17,21]. They affect the relaxation properties of the nuclei they interact with. The mechanism for contrast enhancement is based on the shortening of the T_1_ and/or T_2_/T_2_* relaxation times of the regions in the eye influenced by the contrast agents [8,21]. ^19^F MRI can be used to detect ^19^F-based compounds attached to cells present in regions of interest of the eye or to monitor efficacy of stem cell therapies [17].

An additional way to generate unique contrast in MRI without administering contrast agents is that achieved by the magnetization transfer contrast (MTC) technique. In MTC, coupling between rigid ^1^H nuclei (for example, in macromolecules or membranes) and mobile ^1^H nuclei (in liquids) allows the state of the rigid ^1^H nuclei to influence the state of the mobile ^1^H nuclei through exchange processes. It is possible to magnetically label ^1^H nuclei in ocular macromolecules or membranes preferentially using an off-resonance RF pulse. The rigid ^1^H nuclei have a broader absorption line shape than the mobile ^1^H nuclei, making them as much as 10^6^ times more sensitive to an appropriately placed off-resonance irradiation. This preferential magnetic labeling of the rigid ^1^H nuclei can be transferred to the mobile ^1^H nuclei, depending on the rate of exchange between the two populations of ^1^H nuclei, and hence can be detected with MRI [15].

The eye also contains cells expressing intracellular and cell surface proteins present elsewhere in the body [2]. Several experimental animal models of eye diseases, mimicking eye diseases in humans, have been developed [22]. These diseases can be characterized by changes in the thickness of eye membranes and changes in the shape and extent of retinal detachment [23]. Experimental diseases can be useful models for the evaluation of new MRI techniques for in vivo monitoring ocular diseases or to assess the efficacy of new therapies. The use of high-field multiparametric MRI and spectroscopic methods for in vivo and global assessments of the visual system and the visual cortex of the brain in rodent studies was reviewed by Chan et al. [24].

Changes during inflammatory and immune-mediated diseases, including ocular disease, have been associated with inflammatory macrophages [25]. Ocular MRI may thus be useful for the in vivo evaluation of macrophage-specific MRI techniques. High spatiotemporal resolution and adequate image contrast are required for in vivo investigation of the macrophage-specific activity in the eye. While MRI hardware (for example, surface receiver coils) can be developed to increase the detected SNR and hence improve image spatial and/or temporal resolution, contrast agents not only enhance the MRI image contrast between eye structures but can also be used as cell markers [21]. Animal MRI eye images in research studies are acquired under anesthesia, and hence longer image acquisition times may be used compared to that used in humans. The temporal resolution is especially important for in vivo animal research studies of ocular perfusion and diffusion and in clinical MRI studies. In the context of the current paper, temporal resolution refers to the acquisition time of a particular image (for example, a T_1_-weighted MRI where the eye most likely moves during the time it takes to acquire the image, rendering short acquisition times, i.e., high temporal resolutions, highly desirable) and for one time point in multiple time point functional/dynamic experiments.

In clinical MRI settings, patients are scanned without being anesthetized and in some studies, with the eyes open and fixating at a static object [26-28]. In these clinical scenarios, the MRI images can be affected by motion artifacts due to the movements of the eye, while the effects of head movements can be minimized by fixating the head during the scanning [29]. The effects of blood pulsation and of constant changes in the state of the lens and the size of the pupil are small compared to the effects induced by eye movements [29] and can be neglected.

The main eye movements during fixations of a static object are drifts, tremors, involuntary saccades, and blinking. Prolonged fixations (i.e., fixations longer than 0.5 s) are invariably subject to independent drifts and small involuntary saccades. Drifts are irregular and relatively slow movements of the axes of the eyes [29]. During 1 s, the axis of the eye has been reported to drift along a path of 15 to 22.5 μm [29,30]. The most probable duration of drifts ranges between 0.3 s and 0.8 s, but the mean duration can be dependent on the subject and on her/his physiologic condition. In the case of long fixations, the duration of the independent drifts can sometimes amount to several seconds. In certain diseases the drifts show a definite direction instead of being irregular. This can also happen in healthy volunteers. In these situations a drift taking place predominantly toward one side is corrected by small saccades in the opposite direction. During prolonged fixations, about 97% of the time is given over to drifts and only 3% to saccades. Drifts are always accompanied by tremors—high frequency and low amplitude oscillatory movements of the axes of the eyes [29]. The maximum amplitudes of such tremors are extremely low, approximately 2.25 μm, and their durations do not exceed 0.013 s [29,30]. Involuntary saccades are high frequency and low amplitude oscillations of the eyes. Durations of saccades range between 0.01 s and 0.07 s, their angular amplitudes being less than 15° in 99% of the cases [29]. The average time between blinks is 2.8 s in men and approximately 4 s in women, with the duration of each blink varying from 0.3 s to 0.4 s [29].

The largest spatial displacements of a human eye axis produced by drifts, tremors, or involuntary saccades during fixations of a static object are at least 4.4 times lower than the highest reported spatial resolution of clinical ocular MRI images to date: 100×200×2,000 μm^3^ [31].

Motion artifacts can, therefore, only be induced on ocular MRI images by drifts, tremors, and/or involuntary saccades in special and rare situations, for example, if the eye axis displacements produced are extremely large and of the order of the spatial resolution of the MRI images. In these special situations, during a time of, say, 5 s, which represents a typically high temporal resolution used in clinical eye MRI studies [31,32], a human eye can, most probably and independently, effect six to 17 drifts, at least 71 tremors, and approximately 500 involuntary saccades. Eye blinks represent the most important sources of motion artifacts in clinical eye MRI [26], with a typical frequency of one blink every 2.5 s. Clinically, temporal resolutions of the order of 1 min are more commonplace than the 5 s noted earlier; during this time the average number of eye blinks effected is 20 in men and 14 in women. To reduce the number of eye blinks as much as possible, some researchers prefer to perform clinical ocular MRI investigations with the closed and relaxed uncovered eye [27] and covered eyes [33] of the scanned subject. If both the scanned and the unscanned eyes are covered, care must be taken to compress the eye as little as possible, using a light material (for example, sterile bandages). Extremely long scan times, on the order of 30 min, which represents the maximal time for a clinical protocol, result in dryness of the eyes in experiments involving eye fixation and in considerable discomfort to the patient, and hence these should be avoided in practice [27,33].

The eye movements and the corresponding optimal imaging protocols for use in rat [34], mouse [35], and human [31,32] ocular MRI have been evaluated in different studies. The combination of ketamine/xylazine and pancuronium was found to be most effective in minimizing rat eye movements and maximizing retinal function [34], while for mice, isoflurane was found to be the most effective anesthetic [35], suggesting potential species differences. An optimal protocol suggested by Zhang et al. [31] for high spatial resolution human ocular MRI studies involves stable eye fixation on a target with cued blinks every 8 s during image acquisition. At lower spatial resolutions and hence acquisition times, the scanned human eye [27] or both eyes simultaneously [32,33] may be kept closed during MRI.

## Magnetic resonance imaging techniques without contrast agents

Depending on the ocular condition investigated, achieving high spatial resolution in vivo by MRI is important, especially in cellular-specific MRI studies or when thin human eye structures need to be visualized and quantitatively analyzed. The mean radius of a human macrophage is approximately 20 μm [36]. Mean human corneal thicknesses vary between 800 μm and 1,200 μm, and mean thicknesses of the human sclera range between 400 μm and 1,500 μm [37]. Artifacts induced by eye movements on clinical ocular MRI images can be avoided or reduced as much as possible by acquiring MRI images with temporal resolution as high as possible [26] and by careful patient eye positioning during scanning [27,33].

### Magnetic resonance imaging techniques used to acquire high spatiotemporal resolution eye images

Surface detector coils can be used to achieve high spatial and/or temporal resolution of MRI images in general, including eye images. The best ocular imaging scenario uses a head volume coil in the transmission mode and an eye-shaped single or double loop surface coil in the reception mode [38]. In this way, uniformity of the excitation RF field is achieved with the head volume coil, while the eye-shaped detector coil is used to maximize the SNR in the images. If uniformity of the excitation RF field is sufficient over the eye volume, an eye-shaped transceiver surface coil (that is, a coil that can be used in both transmit and receive modes) may be used to acquire images with even higher SNRs. This increase in SNR can be used to improve the spatial and/or temporal resolution of the images [39]. Eye structures, such as the retina, iris, ciliary body, lens, and aqueous and vitreous humors, of a single human [14,26-28,40], mouse [41,42], and rat [7] eye or of both human [43] and rat [38] eyes can be visualized in vivo using surface coils.

**Figure 1:**
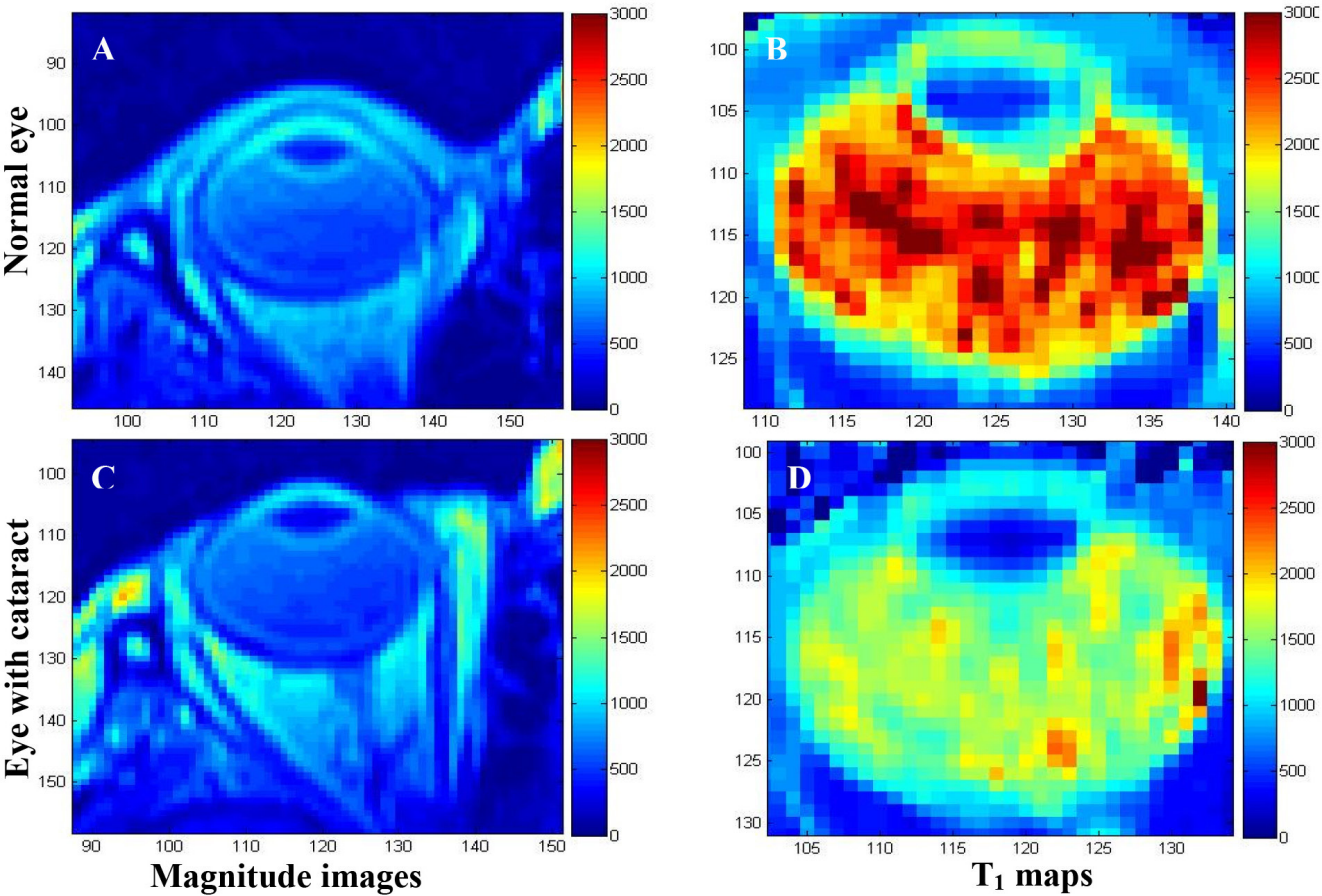
T_2_-weighted magnetic resonance images: **A**, **C** and the corresponding T_1_ maps: **B**, **D:** The image and T_1_ map in panels **A** and **B** are that of a normal eye, while those in panels **C** and **D** are of an eye with cataract. No significant differences were identified between the magnitude images of the normal eye in **A** and that of the eye with cataract in **C**. The T1 values calculated in ms on the T1 maps show that the T1 values of the eye with cataract in **D** were two times lower than that of the normal eye in **B** in the vitreous and aqueous humors. The spatial resolution of the images is 780×1560×2000 μm^3^, and the images were acquired in approximately 1 min. The images were reproduced from [33] with permission from the Romanian Society of Medical Imaging.

**Figure 2:**
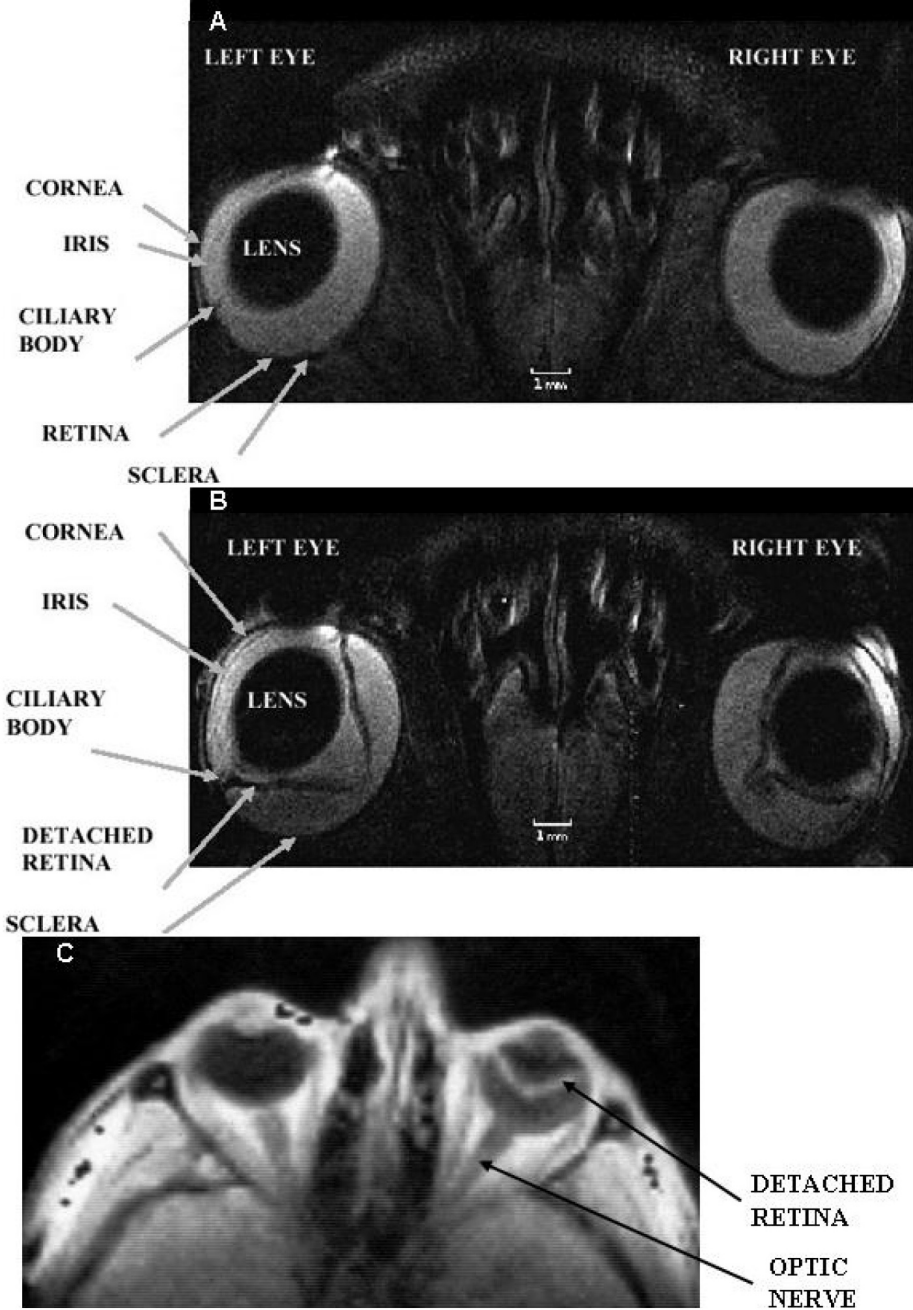
Selected regions of in vivo MRI images of the rat (**A** and **B** and the human **C** eye. Eye structures such as the lens, cornea, iris, ciliary body, retina and sclera are visualized. In **A** both rat eyes are normal. The left rat eye in **B** is at peak of experimental autoimmune uveitis and the right rat eye in the same figure shows post-peak of disease. The spatial resolution of each MRI rat image was 60×60×700 μm^3^ and the acquisition time was 30 min. The left human eye in **C**: shows no retinal detachment, while the right human eye in the same figure shows a large retinal detachment. The MRI images in **A** and **B** are T_2_-weighted, while that in **C** is T_1_-weighted. The images in **A** and **B** were reproduced from [38] while that in **C**: from [43] with permissions from John Wiley & Sons and Elsevier Ltd respectively.

Anatomic information of normal and diseased human eye visualized in vivo by T_2_-weighted MRI at 1 T is shown in Figure 1 [33]. The corresponding quantified T_1_ maps in this figure reveal a greater than twofold decrease in T_1_ values in the vitreous and aqueous humors of the eye with a cataract. Figure 2A,B show the anatomic information of normal and diseased rat eye visualized in vivo by T_2_-weighted MRI at 4.7 T [38]. The images were acquired in 30 min at a high spatial resolution of 60×60×700 μm^3^ and clearly show structures such as the lens, cornea, iris, ciliary body, retina, and sclera. A large human retinal detachment was visualized by Deans et al. [43] using a surface coil in the receive mode (Figure 2C). Retinal abnormalities were also assessed by measuring the thicknesses of the retina on MRI images of the human eye at 1 T [14] and at 3 T [31].

More recently, variations in eye volume, surface area, and shape, with the refractive error in the mouse at 4.7 T [44,45] and human at 3 T [46], were quantitatively evaluated. The authors showed that both the mouse and human vitreous humor are elongated in myopia and that both mouse and human eye shapes change even in the early stages of myopia. The three-dimensional enlargement of normal eyes is uniform, while that of myopic eyes takes place along the axial globe direction only, determining the prolate shape of the myopic eyes [46].

Visualization of human eye structures in vivo requires high spatiotemporal resolution [26,32]. The average diameter of the human eyeball is approximately 25 mm, while that of the rat eye is approximately 5 mm [47]. The eye contains thin eye structures; for example, the maximum retinal thicknesses of normal rat [38], mouse [42], and human [14] eyes measured on ocular MRI images acquired in vivo ranges between approximately 200 μm [38,42] and 1,200 μm [14].

A high spatial resolution clinical ocular MRI study allowed for the measurement of the mean thickness of the human retina/choroid complex, which was found to be 711 μm, and for the identification of three layers in the retina [36]. A recent high spatial resolution ex vivo animal ocular MRI study allowed for the visualization of several layers in the region of the retina/choroid complex, albeit with poor contrast [48].

**Figure 3:**
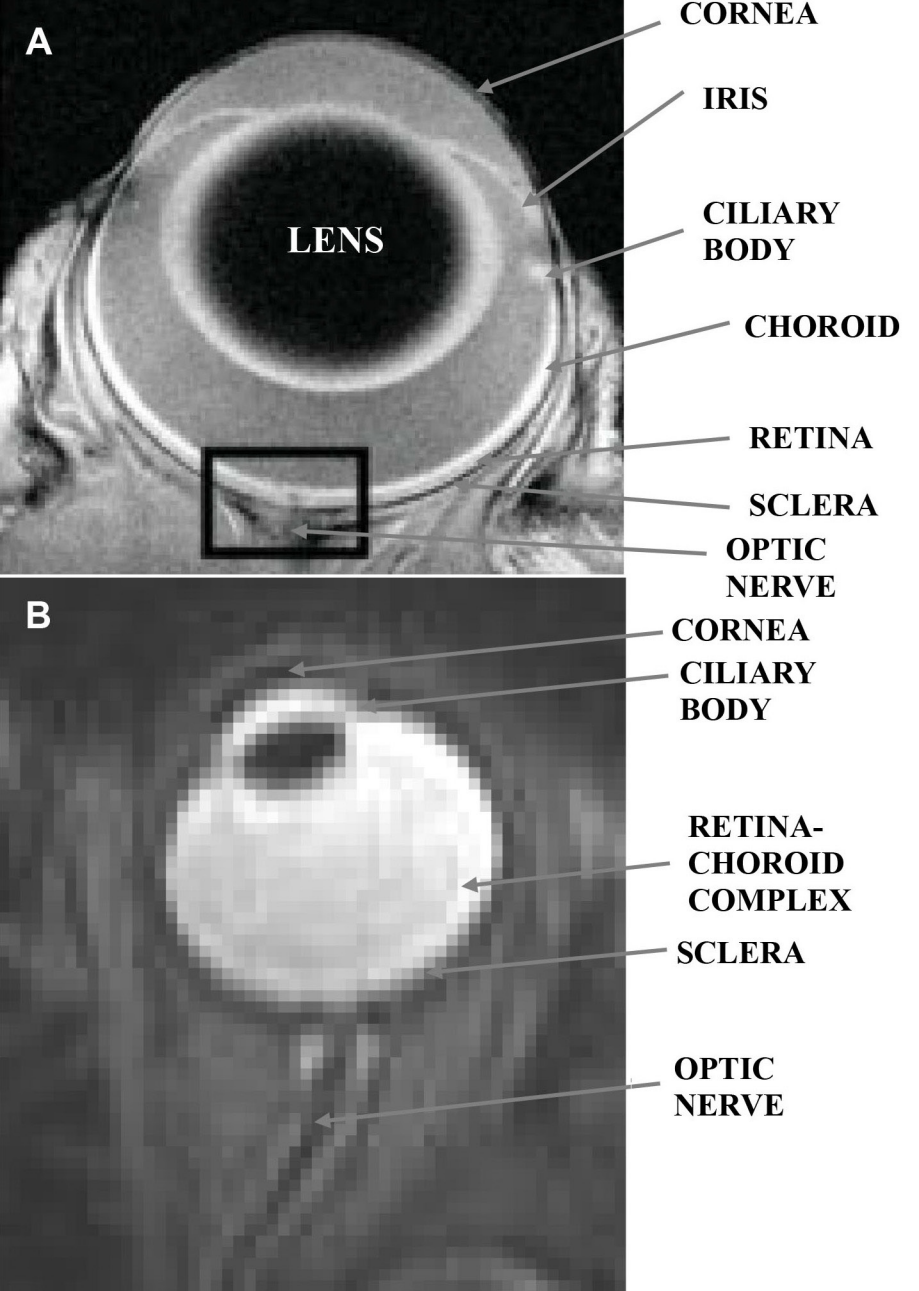
The highest spatial and temporal resolution of in vivo rat: **A** and human: **B**: ocular magnetic resonance imaging images. The spatial resolution of the magnetic resonance imaging (MRI) image in **A** is 23×23×620 μm^3^, and its acquisition time was 50 min. The spatial resolution of the MRI image in **B**: is 780×1,560×2,000 μm^3^, and its acquisition time was 5 s. The MRI image in **A** is contrast agent enhanced, while that in **B**: is T_2_-weighted and no contrast agent was used during its acquisition. The image in **A** was reproduced from [7] with permission from John Wiley & Sons Ltd.

The highest reported in-plane spatial resolution for in vivo ocular multislice MRI images is 23×23×620 μm^3^ for animal studies [7] and 100×200×2,000 μm^3^ for human studies [31], with acquisition times of 50 min and 8 s, respectively. Representative images from these studies are presented in Figure 3. For three-dimensional acquisitions, on the other hand, spatial resolutions as high as 42×42×84 μm^3^ and 20×20×57 μm^3^ were recently achieved in vivo in ocular MRI animal research studies [48,49].

The reduced MRI signal intensity collected from the resultant smaller voxel volumes in these high-resolution images invariably leads to a decrease in the MRI image quality. The MRI image quality may be improved to some degree via signal averaging with the consequent increase in acquisition time. A compromise between acquisition time and image quality always has to be achieved in ocular MRI, given the propensity for the eye to move over short timescales and the small structures present that need to be visualized. One can thus place a realistic upper limit of approximately 2 min on the acquisition time for human ocular MRI images.

**Figure 4:**
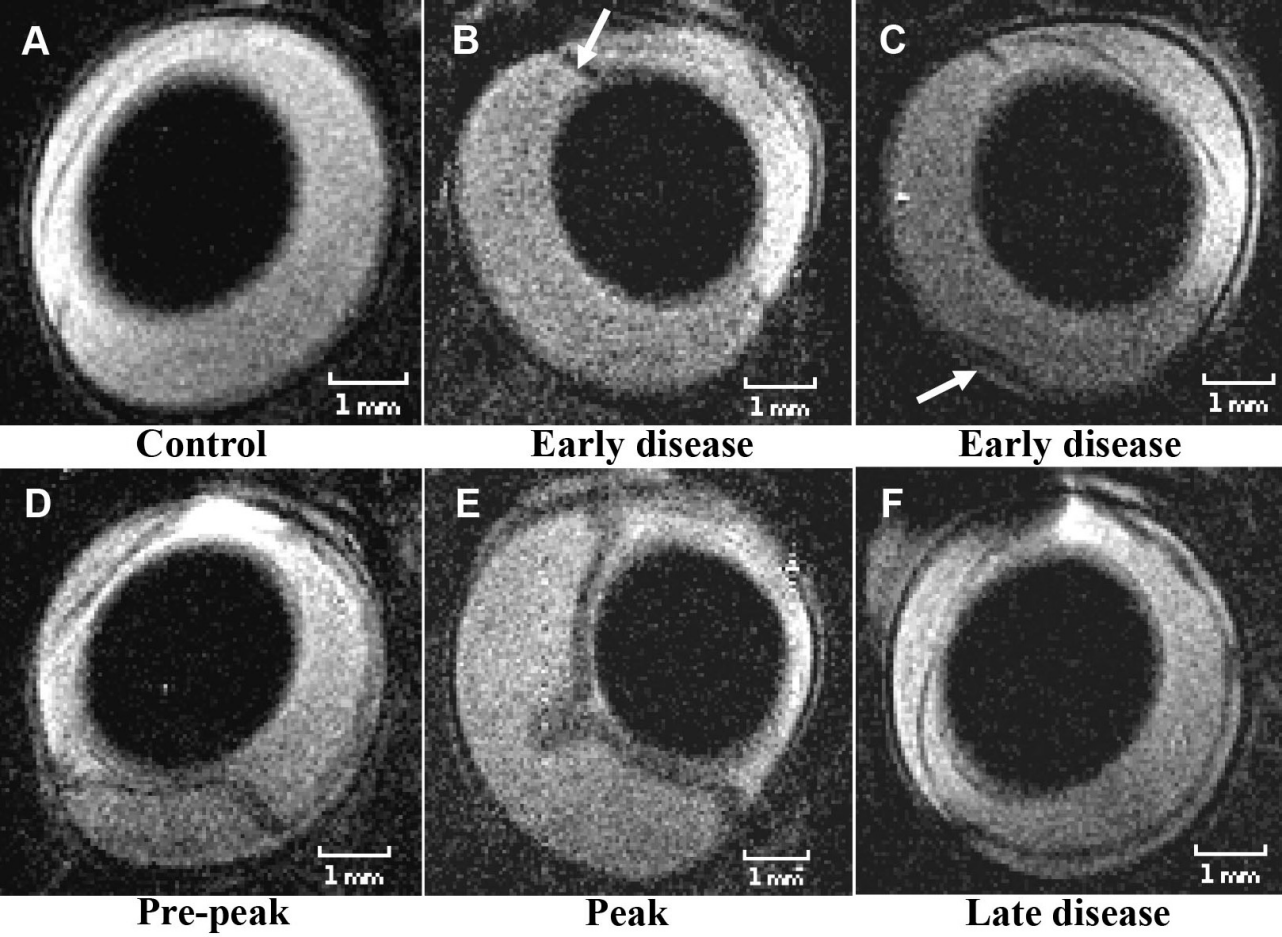
Normal rat eye and rat eyes at the different stages of experimental autoimmune uveitis. The MRI images of the normal: **A** and that of the eyes affected by experimental autoimmune uveitis (EAU): **B**-**F** were acquired in vivo at 4.7 T. The inflamed ciliary body and a small retinal detachment were the first signs of the disease detected in vivo by magnetic resonance imaging (MRI) and confirmed ex vivo by histology. Detection of the inflamed ciliary body using MRI is shown with a white arrow in **B** Inflammation of the ciliary body was confirmed by histology. The smallest retinal detachment detected in vivo using MRI is shown with a white arrow in **C** and confirmed on the corresponding histological section ex vivo. The maximum retinal detachment was detected at the peak and post peak of EAU in vivo using MRI, **E** and confirmed ex vivo by histology. The spatial resolution of each MRI image is 60×60×700 μm^3^, and the acquisition time was 30 min. All MRI images in this picture are T_2_-weighted. The images were reproduced from [38] with permission from John Wiley & Sons Ltd.

The resolution (spatial and/or temporal) can also be improved using specially designed surface coils that can be placed over both eyes, allowing for the simultaneous acquisition of images of both eyes. Figure-eight surface receive-only coil [38] was designed and constructed for just this purpose. The resultant images are presented in Figure 2A,B and Figure 4, where retinal thicknesses as small as 100 μm were measured at 4.7 T [38]. In this study, retinal abnormalities in a rat model of experimentally induced autoimmune uveitis (EAU) were detected in T_2_-weighted images, with findings confirmed ex vivo by histology.

A similar image acquisition setup was used by Deans et al. [43] to acquire rat eye images, which also benefited from the reduced time to acquire images of both eyes.

Histologically ex vivo-confirmed sites of infiltrated macrophages were also detected in vivo by MRI in the region of the retina of rat eyes at the peak of EAU [39]. The MRI images in this macrophage-specific study were acquired using a two-turn single-loop receiver coil placed over one eye of an anesthetized rat, where the second loop was designed to increase the SNR over a single-loop coil [39].

### Blood-oxygenation, level-dependent, functional magnetic resonance imaging techniques

Retinal function and ocular physiology can also be evaluated using fMRI techniques [11]. fMRI can be performed with or without contrast agents and is most commonly used to evaluate brain processes ranging from sensory perception to cognitive functions. The most commonly used fMRI technique is based on blood-oxygenation level-dependent **(**BOLD) contrast. The BOLD contrast is determined by the intravoxel magnetic field inhomogeneities produced by the paramagnetic deoxyhemoglobin in the erythrocytes in the blood. A regional reduction in deoxyhemoglobin concentration is produced due to an increased neural activity when a specific task is performed, for example, when a visual stimulus is presented to the subject. These changes can be visualized and/or quantified on T_2_ and T_2_*-weighted MRI images [11]. Duong et al. [11] acquired BOLD–fMRI eye images during dark, drifting grating, and stationary grating stimuli to evaluate retinal function and physiology in the cat eye. Three different segments in the retina and differential responses in the two vascularized retinal regions were detected [11]. In a separate study, spatial-independent component analysis was applied to the data to quantify the human eye movements and to estimate the point of gaze at the time of data acquisition [50]. Information on the retinal/choroidal blood flow of normal, unanesthetized, and awake humans was also achieved at 3 T using BOLD–fMRI techniques [51].

### Diffusion-weighted and diffusion-tensor magnetic resonance imaging techniques

Diffusion-weighted imaging (DWI) and diffusion-tensor imaging (DTI) can quantitatively assess the isotropic (DWI) and anisotropic (DTI) water diffusion in tissues in vivo [8,9]. DWI uses additional magnetic field gradients to encode diffusion of ^1^H nuclei in water molecules in the tissue investigated. Several MRI images are acquired using T_2_-weighted pulse sequences and diffusion-encoding gradients with at least two different amplitudes. Although it is possible to apply the diffusion-encoding gradients along one direction alone, in general they are applied along three orthogonal directions to sensitize the data to diffusion properties in all directions, with an average across all directions calculated. The resulting data are mathematically modeled using a mono- or bi-exponential model of the diffusion of water molecules in tissue, from which quantitative measurements of the apparent diffusion coefficient may be calculated. Fractional volumes of fast and slow diffusing water components may also be calculated, which are thought to correspond to intracellular and extracellular water diffusion. Assessment of the bi-exponential diffusion component is difficult to perform clinically because it requires acquisition of diffusion-weighted images with high SNR [52]. In DTI, the diffusion-encoding gradients have to be applied along at least six, although often more, different directions, and hence acquisition times tend to be much longer than that of a DWI experiment [52]. The quantitative information extracted from DTI images gives more complex information on the anisotropy of water diffusion, which can be evaluated quantitatively in vivo by the eigenvalues of the diffusion tensor, apparent diffusion coefficients, and fractional anisotropy calculated after mathematically modeling the data acquired in a DTI experiment [52].

Using DWI techniques, apparent diffusion coefficient values in normal cat, rat, and mouse retina were calculated at 4.7 T, 7 T, and 11.7 T [53] and the restricted diffusion in an inflammatory optic neuropathy in a 4-year-old girl with acute visual loss in the right eye [54] was detected. Mono-exponential water diffusion in the inner, middle, and outer normal rat retinal layers were also quantitatively assessed by DWI [34].

DTI techniques allowed detection of three retinal layers and one choroidal layer in normal mouse retina [41]. The internal diffusive pathways of the lens ex vivo in bovine [55] and human [56] and in vivo in mice [41] eyes were also determined using DTI techniques. In these studies, elongated fiber cells extending from the anterior to the posterior suture were detected in the lens cortex of the eyes of each species. Individual lens fiber cells were organized into concentric layers parallel to the lens surface [41,56].

The retinal cell alignment and water diffusion in mice were also assessed quantitatively by DTI, where layer-specific apparent diffusion coefficients and fractional anisotropy were detected in the mice retina [41]. The photoreceptor cells in the central retina, adjacent to the optic nerve, were found to exhibit a well organized spatial distribution that is parallel to the optic nerve axis.

### Ocular relaxometry

The clinical potential of the NMR technique was demonstrated in the 1970s by Raymond Damadian who showed that T_1_ and T_2_ relaxation times of tumors were significantly different compared to those of normal tissue [19]. Recent studies have indicated that quantitative MRI relaxometry could help to optimize anatomic, physiologic, and functional contrast in vivo [34]. During the past 40 years, MRI techniques have developed rapidly, and the T_1_, T_2_, and T_2_* relaxation times of the cat [57], rat [34], and mouse [42] retina and vitreous humor were calculated in vivo at 4.7 T, 7 T, and 11.74 T using MRI. More recently, T_1_ [28,32,33] and T_2_ [32] maps of human eyes were extracted from ocular MRI images. T_1_ maps of a normal and of a disease-affected human eye are shown in Figure 1 [33]. Mean T_1_ and T_2_ values in the region of the aqueous humor, lens, vitreous humor, retina, and sclera in eyes of normal controls and patients at different stages of diabetic retinopathy were also calculated from data acquired at 1 T, with statistically significant differences found between the T_1_ values measured in the region of the lens and aqueous humor [32].

These preliminary human studies have demonstrated the potential of MRI relaxometry for clinical ocular MRI, and it is clear that future studies should focus on the development of such techniques at higher magnetic field strengths, while clinical studies need to be performed on more refined disease stages. The use of more quantitative data in clinical imaging in general, and MRI in particular, would be helpful for disease identification, staging, and for elucidating ocular disease mechanisms.

### Quantification of ocular perfusion using arterial spin-labeling techniques

Tissue perfusion can be quantitatively assessed in vivo using the technique of arterial spin labeling (ASL). ASL techniques involve the rapid acquisition of two MRI images, one of the images magnetically labeling the blood flow in the tissue of interest. Typically, adiabatic RF pulses are used to magnetically label the flowing spins (i.e., the ^1^H nuclei in water molecules in the blood vessels) to ensure uniform labeling. Separation of the arterial, capillary, and venous blood flow is possible if appropriate velocity-encoding magnetic field gradients are applied to the tissue of interest. Labeling of the blood flow can be achieved using continuous or pulsed ASL techniques; the optimal technique remains the focus of much research activity. If the tissue–blood partition coefficient and the spin-labeling efficiency are known, the labeled and unlabeled MRI images and a T_1_ map can be used to calculate blood flow maps of the tissue of interest. Each pixel of these maps will indicate the value of the blood flow expressed in units of ml/g/min [9].

Blood flow maps of the rat retina were obtained at 7 T using a continuous ASL technique [58]. The authors assumed that the tissue–blood partition coefficient in the retina was 0.9, while the mean T_1_ value of the retina/choroid complex was found to be 1.7 s and the spin-labeling efficiency was 0.8 for the ASL technique used.

The measured blood flow in the rat retina/choroid complex and the rat ciliary body were high, whereas that in the rat cornea and rat vitreous humor were essentially absent or within noise levels. Blood flow of the normal rat retina/choroid complex (4–6 ml/g/min) was markedly higher than cerebral blood flow (0.9–1.1 ml/g/min) under essentially identical experimental conditions, including 1.1% isoflurane anesthesia. The blood flow in the rat retina/choroid complex with spontaneous retinal degeneration was markedly reduced compared to the corresponding control values. Blood flow is tightly coupled to basal metabolic activity, so the reduced metabolism of degenerated retinas of rats is expected to lead to reduced blood flow. The reduction of blood flow in the rat retina/choroid complex is in agreement with results in studies on neurodegenerative brain diseases. The reduction of the rat blood flow in the region of the optic nerve head did not confirm the theory of high vascular density in the region of the rat optic nerve head [58]. Blood flow along the retina/choroid complex was relatively uniform (as expected, since rats do not possess a fovea), contradicting the theory that the optic nerve head is densely populated by large arteries and veins in the rat [58].

It was not possible to separate the rat choroidal and retinal blood flow in vivo using the ASL MRI technique described by Li et al. [58]. Other techniques allowed separation of the rat choroidal and retinal blood flow measurement but they involved radioactively labeled microspheres. Based on results obtained using microsphere techniques, the rat choroidal blood flow was about ten times more rapid than either rat cerebral or retinal blood flow [58].

More recently, an improved ASL technique allowed for the separation of the choroidal and retinal blood flow in mice [59,60] and in rats [61]. The choroidal blood flow in the normal mice was found to be five to six times more rapid [59], while in the normal rats this was eight times [61] more rapid than the retinal blood flow. The retinal and the choroidal blood flow decreased in the mice affected by glaucoma [60] and increased in the rats with retinal degeneration [61] compared to the corresponding blood flow of normal mice and rats. In the rats with retinal degeneration, the difference between the choroidal and the retinal blood flow decreased with approximately 25% compared to the difference detected in the normal rats [61], the choroidal blood flow remaining more rapid relative to the retinal blood flow.

Assessment of the blood flow of the human retina/choroid complex is also feasible using ASL techniques. Peng et al. [62] reported for the first time a blood flow of 93 ml/(100 ml min) of normal, unanesthetized, and awake humans. Zhang et al. [63] showed recently that the human retinal/choroidal blood flow increases by 25% during brief handgrip exercises.

The ASL technique can also be set up to be more sensitive to smaller vessels, such as arterioles, capillaries, and venules, if the imaging parameters are adjusted to minimize contributions from large vessels. For example, the inclusion of a delay (for example, 200 ms) between spin labeling and image acquisition allows time for the labeled spins to leave the large arteries and move into the smaller vessels, thereby decreasing the sensitivity of the large arteries. Second, the labeled spins lose their degree of magnetic labeling by the time they reach large draining veins (since the T_1_ of blood is approximately 2 s), thereby decreasing the impact of large veins. Selective labeling of the flowing spins can also be achieved based on their velocity using additionally applied, velocity-encoding, magnetic field gradients. Selectively detecting blood flow in smaller vessels is advantageous since it more accurately reflects local tissue perfusion. It is also interesting to note that, in contrast to most optically based approaches, ASL measures tissue perfusion of labeled water in the whole tissue within a voxel without the need to resolve individual vessels [9,58]. The advantages of the ocular ASL techniques over the optically based ocular imaging techniques are that they have the potential to provide detailed quantitative information on the blood flow and volume over the whole volume of an eye or both eyes simultaneously, with acquisition times on the order of minutes, and do not require transparent media for the propagation of the electromagnetic waves through the eye during image analysis. Arterial, venous, or capillary blood flow can be separated and quantitatively analyzed using ASL techniques. More complex quantitative information can also be extracted from the ASL data using compartmental modeling [64]. However, high spatiotemporal resolution is required to separate measurements of the retinal and choroidal blood flow, while the achievable SNR in in vivo ASL studies tends to be low, rendering it difficult to mathematically model the data to extract accurate quantitative information on the perfusion. In animal studies, the data have been found to be influenced by the type of anesthesia performed, although this will pose less of a problem in clinical studies (of course, problems with eye movement will then arise). Nevertheless, ASL techniques in general, and ocular ASL techniques in particular, need to be improved to boost the achievable SNR before they will become clinically available [9,58].

## Magnetic resonance imaging techniques with contrast agents

Responses of the retina and choroid to different stimuli and perfusion of the blood–retinal barrier (BRB) can both be quantitatively evaluated, while ocular anatomy and cellular activity within the eye can be qualitatively evaluated, via MRI using a variety of extracellular and intracellular contrast agents. High spatial resolution is important when detailed anatomic and physiologic information and/or cellular detection are needed, while high spatiotemporal resolution is needed in dynamic MRI studies.

T_1_- and T_2_-based contrast agents can be used to enhance the contrast between different eye structures [14,65-67]. Perfusion of the blood–retinal barrier can be assessed quantitatively using dynamic contrast-enhanced (DCE) MRI [65,66] and dynamic susceptibility contrast (DSC) MRI [67] techniques or using gaseous contrast agents [58,67-69]. Although most clinical studies involve imaging ^1^H, ^19^F MRI can also be performed in vivo [70]. MRI techniques may also be used to evaluate disease mechanisms [17,39,65,66,68,70,71], to monitor in vivo the cellular activity within the eye [39], or to evaluate response to regenerative therapies using stem cells [70]. Currently, a minimum of 2,000 labeled cells is required for ^19^F cell-trafficking detectability versus the single cell possibility with iron oxides ^1^H MRI [71].

### Intravascular contrast agents

Extracellular contrast agents based on gadolinium (Gd), manganese (Mn), and superparamagnetic iron oxide (SPIO) nanoparticles have been used in several ocular MRI studies. Gd-based agents are commonly used in clinical MRI to enhance image contrast to improve visualization of anatomic information and also for the evaluation of the diffusional pathways of plasma-derived solutes through the blood-to-ocular barriers [72-74] or in dynamic contrast-enhanced MRI studies [65].

Only one Mn-based agent has been approved for human use, and thus most of the published literature is restricted to animal imaging studies [7,75-83]. It is clear that further investigations need to be performed on these agents before widespread clinical acceptance will be achieved.

#### Ocular anatomy, physiology, and pathology assessed using gadolinium-diethylene triamine pentaacetic acid

Bahn et al. [14] demonstrated that retinal thicknesses of the human eye larger than 1,200 μm measured on Gd-*diethylene triamine pentaacetic acid* (DTPA)-enhanced T_1_-weighted MRI images reveal the presence of pathology not detectable on noncontrast-enhanced images. Significant changes in signal intensity were detected in the region of the retina of the disease-affected human eye on images acquired using turbo fluid-attenuated inversion recovery with Gd-DTPA. Nine different layers were identified in the region of the retina/choroid complex on Gd-enhanced high spatial resolution MRI images of rat eyes acquired ex vivo [48].

Contrast agent-enhanced ocular MRI techniques were also used to evaluate the diffusional pathways of plasma-derived solutes in the vitreous and aqueous humor through normal human [74] and disease-affected rat [72,73] blood-to-ocular barriers. These evaluations showed that the plasma-derived solutes that flow into the anterior chamber of the normal human eye are combined with a predominantly unidirectional flow of aqueous humor anteriorly through the pupil. The tight junctions of the iris epithelium prevent the nonspecific diffusion of agents into the posterior chamber of the normal human eye [74]. The rat studies involved an investigation of the etiology of biochemical changes in the glaucomatous chamber of the eyes [73] and the mechanisms of protein infiltration into the vitreous humor produced before the development of neovascularization in newborn rats affected by the retinopathy of prematurity [72]. The former study showed that the increased permeability of the blood-to-aqueous or aqueous-to-vitreous barrier represents the sources that may implicate the cascades of neurodegenerative processes in the glaucomatous rat retina and optic nerve.

#### Dynamic contrast-enhanced magnetic resonance imaging techniques using gadolinium-based contrast agents

DCE MRI is a clinically applicable technique that does not require a clear optic medium. It involves intravenous injection of a Gd-based contrast agent and the acquisition of T_1_-weighted MRI images with high spatiotemporal resolution before, during, and after injection of the contrast agent. Injection of the contrast agent can be fast (bolus injection) or slow. Analysis of DCE data are performed using either a shape analysis of the Gd concentration time curves (for example, looking at time to peak, area under the curve at 60 s, etc.) or via a pharmacokinetic modeling of the underlying tissue and fitting these models to the concentration time curves, depending on the type of infusion used [8-10].

Two-dimensional maps of the influx of the contrast agent in the vitreous humor from the retina, iris, or ciliary body can be obtained by postprocessing the ocular DCE MRI images. The surface area product was also calculated to quantify blood–retinal barrier permeability in normal and diabetic rats [65].

#### Manganese-enhanced magnetic resonance imaging techniques using manganese-based contrast agents

The neuronal connectivity between the eye and brain is important for maintaining the performance of the mammalian visual system [24] and for investigating mechanisms of ocular diseases [76]. A major obstacle in the research of developmental processes and new treatments for neurodegenerative diseases in the visual system is the lack of a precise and sensitive technique for directly assessing the spatiotemporal evolution of the visual pathways in longitudinal studies. Although recent studies showed that treatment to both the eye and the brain for ocular diseases may result in better outcomes than treating the eye alone, current diagnoses of human ocular diseases and injuries are generally limited to the anterior visual pathway [24]. Few methods, including behavioral, electrophysiological, and positron emission tomography, have the ability to evaluate both retina and brain in the same animal and session with high resolution [77]. Mn-enhanced MRI (MEMRI) is a promising approach for addressing this limitation since it can measure function and structure of the retina or brain with high spatial resolution.

Therapies applied to the posterior segment of the eye using eye drops are inefficient, while drug administration using intravitreal and periocular injections are toxic and may lead to complications and patient discomfort [78]. Iontophoresis is a method used to deliver a compound across a membrane by the assistance of an electric field and has been extensively studied in other routes of drug administration in rabbits [78,79]. Efficacy of transscleral [78,79] and transcorneal [78] iontophoresis for the application of therapies to the posterior segment of the eye can be evaluated using MEMRI. Dynamic MEMRI provides an in vivo, quantitative, longitudinal, and three-dimensional method to investigate normal eye adaptation to light and dark conditions [66] or to assess abnormalities in the visual components of rat models of chronic glaucoma [76].

MEMRI has been used as a neuronal tract tracer for several neuronal pathways, including the visual pathway, in a variety of animal models. MEMRI has three primary applications in biologic systems: contrast enhancement for anatomic detail, activity-dependent assessment, and tracing of neuronal connections or tract tracing. Contrast in MEMRI is achieved based on the following main effects produced by the paramagnetic Mn^2+^ ions: T_1_ shortening of tissues where they accumulate and ability to enter excitable cells via voltage-gated calcium (Ca^2+^) channels based on their properties of Ca^2+^ analogs. Once in the cells, Mn^2+^ ions can be transported along axons by microtubule-dependent axonal transport and can also cross synapses transsynaptically to neighboring neurons.

Several MEMRI methods applied to biologic systems, including the visual pathway, were recently described in detail by Massaad et al. [80]. A novel theoretical model of the axonal transport of Mn^2+^ ions was experimentally validated in the rat optic nerve by Ølsen et al. [81]. The efficacy of the topical [82], intravitreal [78,79,81,82], transscleral or transcorneal iontophoresis [78,79], intravenous [66], or intraperitoneal [83] administration route of Mn-based MEMRI contrast agents for different species has also been investigated. Administration routes were evaluated by several authors for functional [66,76,84,85], functional and structural [84], and dynamic [66,76] MEMRI and for the study of the kinetics of Mn^2+^ ions in the eye [81] or of the feasibility of integrative functional cerebroocular [77,78] MEMRI.

#### Functional ocular manganese-enhanced magnetic resonance imaging

The retinal function in normal and disease-affected mice eyes was evaluated by MEMRI in several studies [66,76,84,85]. Relative MRI signal intensity measurements were used to evaluate the regional retinal ion regulation in vivo and to investigate light-dependent rat retinal melanopsin-induced activity, using MEMRI. Greater Mn accumulation was found in the light-adapted retinas of wild-type and knockout mice compared to dark-adapted wild-type mice and knockout mice [84]. The sensitivity of MEMRI to regional light responses in the mice retina was also evaluated by Ivanova et al. [85]. High spatial resolution MEMRI also revealed lamina-specific neurodegenerative changes of the retinal structures in retinal diseases and allowed visualization of seven distinct Mn contrast-enhanced layers in the region of the normal rat retina [86]. More work is needed to analytically evaluate how the spatial distribution and extent of retinal Mn uptake are linked, for example, with light exposure frequency and intensity [86].

#### Functional and structural ocular manganese-enhanced magnetic resonance imaging

The imaging of a human choroidal melanoma xenograft transplanted into the eye of a nude rat permitted the simultaneous structural evaluation of the tumor and the neighboring retina in the same eye using MEMRI [83]. Mn^2+^ was taken up by the tumor, which resulted in contrast enhancement during MEMRI imaging. The retina in the inferior portion of the eye, which was not in direct contact with the tumor, was adversely affected by the presence of the melanoma. Inferior outer retinal MEMRI intensity was altered in those eyes, and the inferior retina was edematous. The use of the MEMRI technique in this choroidal melanoma model permitted the study of the impact of tumor growth on the neighboring retina and allowed the simultaneous evaluation of treatment-related side effects in the same eye. Although one Mn-based contrast agent (Teslascan) recently received FDA-approval in the USA and motion artifact-free high-resolution MRI images of the human retina can routinely be collected, the clinical usefulness of this MEMRI study for monitoring choroidal melanoma progression and/or treatment response in patients has not yet been evaluated [83].

#### Dynamic ocular manganese-enhanced magnetic resonance imaging

Dynamic ocular MEMRI findings and their confirmation by histology allowed association of the progression in time of experimental chronic glaucoma induced in rats with retinal ganglion cell loss, axonal density decrease, and/or disturbance of fast axonal transport. Mn^2+^ transport rates at the prechiasmatic optic nerve of approximately 3.02 mm/h were estimated for normal rat eyes, while the corresponding rates estimated for rat eyes affected by chronic glaucoma were reduced. These reductions in transport rates may be caused by several factors: retinal ganglion cell loss, a significant reduction in the average axonal densities at the prechiasmatic optic nerve, or obstruction of the glaucomatous optic nerve. Accumulation of Mn^2+^ ions in the vitreous humor of the glaucomatous rat eye was possibly produced by perturbation of the usual pattern of Mn^2+^ clearance in the glaucomatous eyeball. High concentrations of Mn^2+^ ions were also observed at the optic nerve head and the retina [76].

Complex quantitative information can be extracted from dynamic MEMRI data, using tissue compartment models. Retinal transfer rates can be estimated using least-square fittings of the MRI data to the compartment model that is assumed. Based on these fittings, on the relaxivity of the contrast agent used, and on the T_1_ value of the eye structure evaluated (before injection of the contrast agent), transfer rates of an agent through the retina were estimated to be as small as 0.5×10^−3^ l/min in the rat eye, while the intraretinal function was also evaluated [66]. These analyses allowed for the differentiation of retinal segments into inner and outer eye segments. The Mn-based contrast agent used is FDA approved in the USA, but further studies are needed to evaluate its clinical usefulness [66].

#### Quantitative evaluation of Mn^2+^ kinetics in the eye

Changes in the entrance and transport rate of axonal Mn^2+^ ions can be used to reveal new information about how white matter disease affects axonal transport. Ølsen et al. [81] developed three different theoretical models for Mn^2+^ ion kinetics in the eye and evaluated them experimentally in vivo in the rat eye using MEMRI. Mn^2+^ ion intracellular storage in the retina caused by restricted Mn^2+^ ion vesicle packing was observed. The degree of Mn enhancement in the optic nerve was found to depend not on the dose injected but on the duration of which Mn^2+^ was available from the vitreous body. The retinal ganglion cell axon entrance of Mn^2+^ ions was found to be rate dependent and not directly proportional to the vitreal concentration, suggesting that increased Mn enhancement could be achieved using a slow-release contrast agent without reaching toxic levels of local concentrations. This study also revealed that transport of Mn^2+^ ions does not depict synaptic vesicle transport rates directly [81].

#### Feasibility of integrative functional cerebroocular manganese-enhanced magnetic resonance imaging

The feasibility of integrating functional measurements from the retina and brain by MEMRI was evaluated in normal neonatal rats [87]. Contralaterally and ipsilaterally projecting axons of the retinal ganglion cells in early postnatal brains, reorganization of retinal and visual callosal pathways upon early blindness, and differential transport mechanisms in developing retinal pathways were also assessed by MEMRI [87].

The target sites of ion delivery and the electric current pathways during transscleral and transcorneal iontophoresis were determined and compared to intravitreal injections and passive delivery of drugs using MEMRI in a rabbit model in vivo. Distributions of the probe ion after iontophoresis, intravitreal injection, and passive delivery were also monitored with MEMRI. These studies should improve the understanding of ocular iontophoresis and help in the optimization of ocular iontophoresis for site-specific drug delivery into the eye [78].

#### Dynamic susceptibility contrast magnetic resonance imaging using superparamagnetic contrast agents

DSC MRI involves the serial acquisition of images before and after an intravenous injection of a contrast agent. As the contrast agent traverses through tissue, the MRI signal intensity is altered due to changes in the T_1_, T_2,_ and/or T_2_* relaxation times of ^1^H nuclei in water molecules in the tissue of interest. The basic concept of the DSC MRI method is to compare transient changes in the concentration of an injected contrast agent in the tissue and its feeding artery of the investigated region. Based on the specific protocol used to acquire the DSC images before and after the administration of the contrast agent and on the mathematical model used to extract the quantitative information from the data, blood volume, blood flow, and vessel size index can be calculated for the region of interest or on a pixel by pixel basis [88].

A superparamagnetic contrast agent and a T_2_*-weighted imaging protocol were used to calculate the ocular blood volume of normal and disease-affected rat eyes. The ratio of choroidal:retinal blood volumes in rats was approximately 10 in the normal eyes, in good agreement with results obtained using different techniques. Based on blood volume measurements, two vascular layers corresponding to rat choroid and retina were identified, separated by an avascular region. The thickness of normal rat choroid/avascular layer/retina was approximately 80/200/60 μm, which decreased to approximately 70/100/50 μm in the rats affected by retinal degeneration. Nair et al. [67] demonstrated that DSC MRI techniques can be used for the quantitative evaluation of layer-specific blood volume in the rat retina, providing important insights into retinal and choroidal hemodynamic regulation in the normal and diseased retina/choroid complex in vivo.

#### Ocular ^1^H magnetic resonance imaging using superparamagnetic iron oxide contrast agents

SPIO-MRI techniques give simultaneous global information about the ocular blood circulation. SPIO-MRI techniques have, therefore, applications in early detection and longitudinal monitoring of retinal diseases, such as retinal ischemia, glaucoma, diabetic retinopathy, and retinitis pigmentosa [89].

Perturbation of retinal and choroidal hemodynamics and neurovascular coupling in different diseases and at various disease stages can be quantitatively assessed by calculating the blood volume and blood flow from MRI images acquired pre-injection and postinjection of the SPIO-based contrast agent. The SPIO-MRI techniques also have clinical potential, with several SPIO-based contrast agents for MRI being approved for clinical use [89].

Visually evoked rat retinal and choroidal responses and rat retinal and choroidal blood circulation patterns were detected using monocrystalline iron oxide nanoparticle (MION) fMRI techniques [89]. In this study, the sensitivity of MION-fMRI to detect the rat retinal response to a stimulus consisting of a 10-Hz achromatic flicker was compared to that obtained with BOLD-fMRI. Although the BOLD signals from the whole rat retina increased during stimulation, its overall sensitivity was about half that of the MION technique at a dose of 30 mg/kg, while the BOLD technique was less reliable in delineating the rat retinal and choroidal vascular responses [89].

Shih et al. [89] also showed that the rat retinal T_2_* response after injection of the SPIO contrast agent is greatest at the optic disc region. The T_2_* responses after injection of the SPIO contrast agent showed that the rat choroid is largely unresponsive to flicker stimulations and the modulation of flicker parameters. Blood volume and flow were also determined based on the difference of the T_2_ and T_2_* relaxation times measured on the MRI images before and after injection of the SPIO contrast agent [89].

### Extravascular contrast agents for ocular magnetic resonance imaging

One of the earliest applications of nanotechnology in MRI involved the use of iron-oxide particles. Iron-oxide crystals have long been used as superparamagnetic T_2_* contrast agents for MRI. SPIO (particle diameter >50 nm)-based contrast agents for MRI are intravascular, while ultrasmall SPIO (USPIO, particle diameter <50 nm)-based contrast agents for MRI are extravascular. For in vivo applications, the particles have nonstoichiometric microcrystalline magnetite core(s) and are typically coated with dextran (e.g., ferumoxide) or siloxane (e.g., ferumoxsil) [21]. Chromium (Cr)-based contrast agents for MRI represent another type of extravascular contrast agent [90].

While at low doses, circulating iron oxides can decrease the T_1_ time of blood and are used in MRI angiography, at the usual doses used for molecular imaging, the T_2_* effects predominate, resulting in marked signal loss on T_2_*-weighted images. Unfortunately, for molecular imaging applications, persistent T_2_* effects from circulating iron oxide nanoparticles make it necessary to delay the MRI examination by 24 h to 72 h postinjection, complicating clinical implementation of these techniques. Iron oxides concentrated at a target site generate magnetic susceptibility artifacts that can extend over the region infiltrated by them, appearing as areas with decreased signal intensity on T_2_*-weighted images [21].

Cr-based contrast agents have also been developed and used as extravascular agents in ocular MRI studies. Cr, specifically hexavalent chromium [Cr(VI)], in the form of potassium or sodium dichromate, is thought to alter heterogeneous tissue oxidation by diamagnetic dichromate [Cr(VI)], which creates paramagnetic Cr species [Cr(V) and Cr(III)] that shorten the T_1_ of surrounding water and/or lipid protons. Structures in the tissue infiltrated by the Cr-based contrast agent will, therefore, appear bright on T_1_-weighted MRI images compared with tissue regions without the Cr-based contrast agent. The retina has a unique lipid profile with the highest level of long-chain polyunsaturated fatty acids in the body and is unmyelinated in most mammalian species but has an uneven distribution of oxidizable lipid contents across layers. The cone and rod outer segments in the outer retina in rodents have the highest lipid density among retinal layers and have many structural similarities as in myelin. Cr-based contrast agents have, therefore, high potential in differentiating retinal layers based on the Cr-based contrast agent uptake and on the T_1_ alteration produced by the contrast agent in the retinal layer [90].

Both USPIO- and Cr-based contrast agents are toxic and thus can be used only in animal research studies [90]. Further developments of these contrast agents are needed before such contrast agents for MRI can be used clinically.

#### Macrophage-specific ocular ^1^H magnetic resonance imaging using ultrasmall superparamagnetic iron oxide contrast agents

Macrophages play an important role during development of autoimmune diseases, including autoimmune ocular diseases. They can be visualized in vivo using high-resolution T_2_-weighted MRI techniques with macrophage-specific intracellular contrast agents [39].

**Figure 5:**
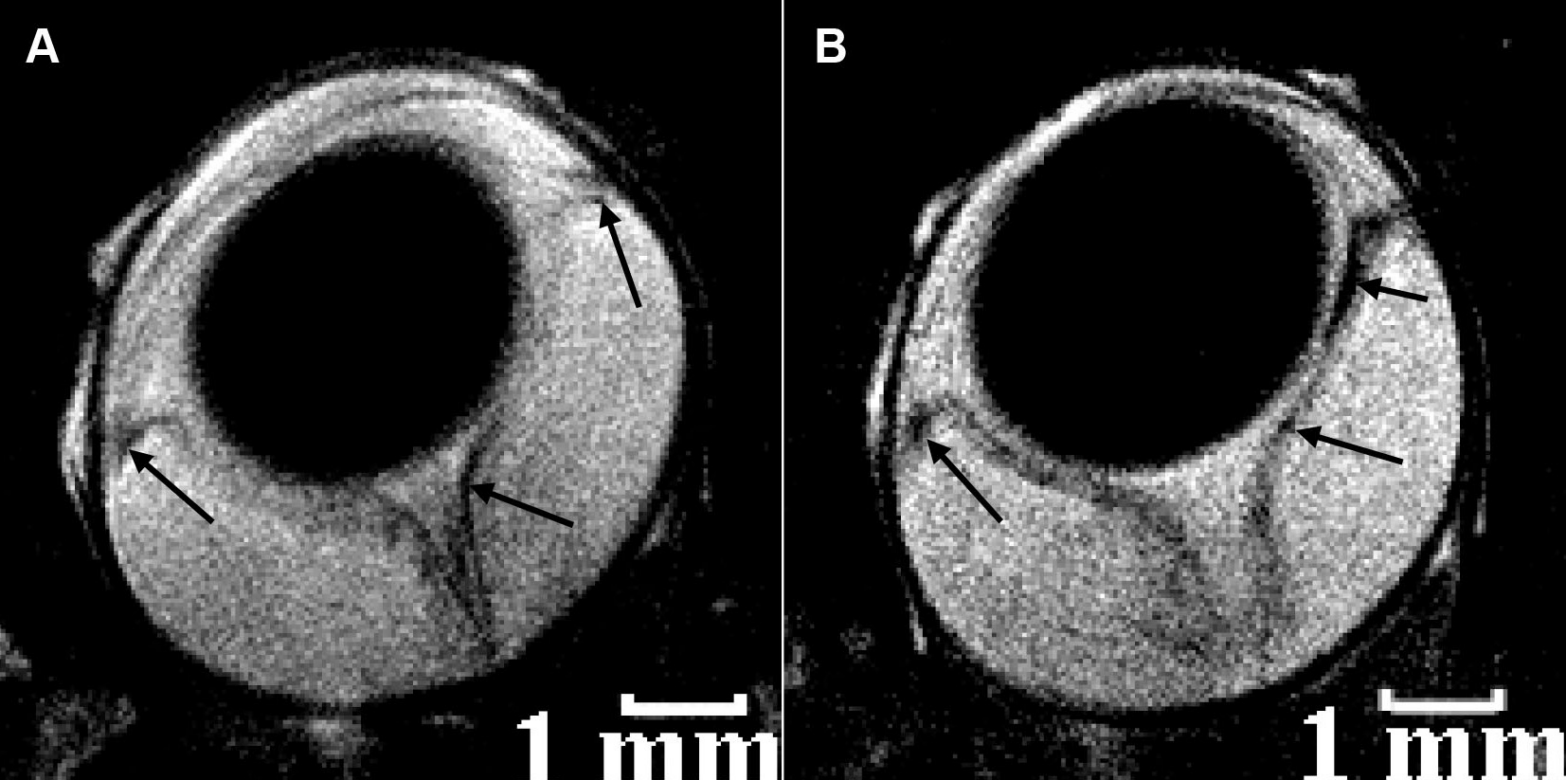
Selected in vivo magnetic resonance imaging transverse sections of rat eyes at the peak of experimental autoimmune uveitis. Sites of decreased signal intensity (arrows) in the region of the retina, iris, and ciliary body are clearly visible. The rats were injected intravenously in the tail vein 4 h before magnetic resonance imaging (MRI) with **A** 0.9% physiologic saline and **B** 300 μM Sinerem solution in 0.9% physiologic saline. The spatial resolution of both MRI images is 40×40×700 μm^3^, and their acquisition time was 25 min. All MRI images in this figure are T_2_-weighted. The images were reproduced from [39] with permission from the International Society of Magnetic Resonance.

A USPIO-based contrast agent and a two-turn single-loop surface coil were used for macrophage-specific MRI of the rat eye affected by EAU, an autoimmune inflammatory disease [39]. Infiltration sites of macrophages in the detached retina of the eye affected by the disease were detected in vivo on MRI images acquired with or without the contrast agent, as shown in Figure 5 [39], and were confirmed ex vivo by histology. The contrast agent used was not clinically approved, but development of new macrophage specific contrast agents might move this study closer to clinical applications.

#### Chromium-enhanced ocular magnetic resonance imaging

Enhancement of the MRI signal in the region of the rat retina on T_1_-weighted MRI images was maximized between 1 h and 3 days and dropped at 1 week and 2 weeks post-Cr(VI) injection. The MRI signal intensity was also enhanced in the region of the rat vitreous humor on T_1_-weighted MRI images at 1 h but unbound Cr was cleared from the vitreous humor within 12 h after intravitreal Cr injection. Higher spatial resolution images (50×50×50 μm^3^ voxel size) were acquired ex vivo and allowed visualization of five different Cr-labeled layers in the region of the rat retina. Significant signal averaging was used to achieve reasonable SNR at this higher spatial resolution, resulting in an acquisition time of 16 h [90].

### Ocular ^1^H functional magnetic resonance imaging using gaseous contrast agents

Rat ocular blood flow has been quantitatively evaluated by fMRI in several studies during hyperoxic (100% O_2_) and hypercapnic (mixture of O_2_ and CO_2_ in different concentrations) conditions, with the air breathing condition used as a baseline in each case. The blood flow calculated in the avascular rat eye structures was extremely low compared to that calculated in the vascular rat eye structures. Hyperoxia was found to decrease the blood flow due to vasoconstriction, whereas hypercapnia increased blood flow due to vasodilation. Rat blood flow in the retina was significantly diminished in degenerated retina and robust hypercapnia- and hyperoxia- induced blood flow changes were observed in normal retinas. Hyperoxia/hypercapnic breathing reduced/increased the total blood flow in the retina of normal animals by 12% and 14%, respectively. Basal blood flow in disease-affected rat retinas was markedly reduced compared to those of control rat retinas. Blood flow is tightly coupled to basal metabolic activity and the reduced metabolism of degenerated retinas of diseased rats may have caused the reduction of retinal blood flow. Hypercapnia- and hyperoxia-induced absolute blood flow changes were not statistically different between normal and diseased rat retinas [58].

Two distinct laminar signals corresponding to the retinal and choroidal vascular layers bounding the retina, separated by the avascular layer in between, were detected based on blood volume measurements. The choroidal blood volume was about ten times larger than the retinal blood volume. fMRI based on blood volume measurements also detected vascular layer-specific responses to physiologic challenges and revealed that choroidal blood vessels were less responsive to physiologic challenges than retinal vessels, indicative of differential hemodynamic regulation of the two vasculatures. In an accepted animal model of photoreceptor degeneration, MRI confirmed the disappearance of the outer nuclear layer and photoreceptor segments. The baseline blood volume values in the retinal and choroidal vasculatures were elevated compared to age-matched controls [67].

The literature on hypercapnic responses and on basal blood flow in the retina is inconsistent, placing these animal studies some distance from likely clinical implementation [67-69]. Despite these inconsistencies, a more recent study on human retinal/choroidal blood flow under hypercapnic conditions (5% CO_2_ and 95% O_2_ inhalation) showed a 12% increase of retinal/choroidal blood flow relative to air inhalation conditions [62].

### Fluorine magnetic resonance imaging

^19^F MR spectroscopy experiments have been performed in the eye [91] and in aqueous humor assay [92]. However, ^19^F techniques are not sensitive enough for imaging in vivo due to the low signal from the significantly lower concentration of ^19^F nuclei in the body compared to ^1^H; hence, only localized ocular ^19^F NMR spectroscopy of large voxels can be used to detect the presence of ^19^F nuclei. In addition, broadband capabilities, which are necessary to detect NMR signals at frequencies other than that for ^1^H, are not common features on most clinical MRI systems. Even if a clinical scanner is equipped with a broadband RF channel for nonproton MRI/magnetic resonance spectroscopy, some hardware development is generally required, such as developing a suitable RF coil [70].

## Future directions in ocular magnetic resonance imaging

Among the MRI techniques for ocular imaging in research and/or clinical environments described in the previous sections, two additional MRI techniques, which do not require the use of contrast agents, show some promise for this field. These techniques are magnetic transfer contrast and magnetic resonance elastography (MRE).

### Ocular magnetic resonance imaging using magnetization transfer contrast

Clinical MRI images mainly ^1^H nuclei in water molecules. The ^1^H NMR signals can be emitted by mobile ^1^H nuclei in water molecules, by less mobile nuclei in lipids, or by rigid ^1^H nuclei in the tissue macromolecules or membranes. The signal emitted by the rigid nuclei is not easily observed directly because of its large bandwidth relative to the free water ^1^H nuclei. However, ^1^H nuclei in the rigid pool can be selectively labeled by applying an off-resonance RF pulse. If the ^1^H nuclei in the mobile and rigid pools are interacting through magnetization exchange (dipolar and/or chemical exchange), then the magnetic labeling of ^1^H nuclei in the rigid pool (macromolecules, membranes) can be transferred to the ^1^H nuclei in the mobile pool (water). Effective coupling between the ^1^H nuclei in the two pools is visualized on the MRI image by a decrease in signal intensity [15]. It has previously been shown that this effect is dependent on the concentration, mobility, macromolecules, or membranes and remodels macromolecular structures within the tissues [93]. The magnetization exchange between the ^1^H nuclei in the two pools can be quantified by calculating magnetic transfer ratios on the acquired MRI images.

Lens opacifications must be accompanied by alterations in tissue compositions, including the hydration state. MTC-enhanced MRI has been successfully used to document lens changes in longitudinal studies of galactosemic dogs during sugar cataract formation [94]. These MTC-weighted images were consistent with localized osmotic lens changes during cataract formation and consistent with the osmotic hypothesis of sugar cataract formation. MTC-weighted images were able to show lens changes sooner than standard MRI images. Measurements of the signal intensity on the MTC-enhanced MRI images revealed that significant tissue changes occurred before any clinically visible lens changes [93]. These measurements also showed that the addition of an MTC preparation pulse to a standard MRI sequence yields high contrast in the dog [94] and human [93] lens. Cortical lens changes were better detected with unenhanced MRI, whereas increased sensitivity to nuclear changes in the lens was observed with MTC enhancement.

MTC-enhanced MRI was also used to quantitatively evaluate the changes produced in the optic nerve in patients with optic neuritis. The quantitative evaluation of the optic nerve using MTC-enhanced MRI may also be useful in the investigation of multiple sclerosis. Magnetic transfer ratio values measured on MRI images in the region of the optic nerve of patients with optic neuritis were significantly different than that measured in corresponding regions of normal eyes [95]. The pathophysiological basis of MTC has not yet been elucidated, and consequently more clinically relevant conclusions cannot be drawn from these studies [95].

### Magnetic resonance elastography of the eye

The mechanical properties of the eye and its anatomic components are believed to be of central importance in many pathologic conditions, such as age-related macular degeneration, glaucoma, Graves’ disease, and myopia. The accurate quantification of these mechanical properties can thus be used to improve the basic understanding of eye mechanics and to enhance patient care, diagnosis, and management of disease [96].

Historically, the mechanical properties of the eye have been a challenge to assess and have proved difficult to quantify, especially noninvasively. Ocular elasticity has been called one of the most confused areas of ophthalmology. The eye comprises multiple tissue types and various anatomic structures that exhibit characteristics of heterogeneity, nonlinearity, anisotropy, viscoelasticity, physiologic accommodation, and extreme sensitivity to hydration—mechanical characteristics that are difficult to account for with a single measurement technique [96].

Conventional mechanical testing methods, including axial strip testing and dynamic mechanical analysis, have been applied to various tissues of the eye, although these techniques are generally limited in their accuracy because testing requires dissection and flattening of doubly curved tissue samples. In addition, these methods are restricted to research applications due to their invasive nature [96].

More recently, experimental image-based approaches for studying eye mechanics have been employed with some limited success by directly measuring displacements (strain) in the eye under inflation conditions. These approaches include techniques such as two-point spot scanning, holographic interferometry, optical imaging of treated corneal surfaces, wave speed analysis using ultrasound, and MRE. Of these techniques, ultrasonography and MRE are the only viable techniques for in vivo applications [96]. MRE is a rapidly developing technology for quantitatively assessing the mechanical properties of the tissue. The technology can be considered to be an imaging-based counterpart to palpation, commonly used by physicians to diagnose and characterize diseases. The success of palpation as a diagnostic method is based on the fact that the mechanical properties of tissues are often dramatically affected by the presence of disease processes, such as cancer, inflammation, and fibrosis. MRE obtains information about the stiffness of tissue by assessing the propagation of mechanical waves through the tissue with a special MRI technique. The technique essentially involves three steps: generating 50–500 Hz shear waves in the tissue using an external driver, acquiring MRI images depicting the propagation of the induced shear waves, and processing the images of the shear waves to generate quantitative maps of tissue stiffness called elastograms [97].

The acoustic shear waves in the tissue are generated by external driver devices. The electrical signal for these devices is created by a signal generator triggered by and synchronized to the MRI pulse sequence and is amplified by an audio amplifier before being fed into the mechanical driver. Over the years, several driving mechanisms have been developed, each with their own advantages and limitations. Spin-echo, gradient-recalled echo, steady-state free precession, and echo planar imaging-based pulse sequences can be designed to be sensitive to motion with a specific frequency, a particular multiple of the specific frequency, or a broad range of frequencies [97]. The resulting tissue motion is encoded into the phase of the MRI images with a synchronous gradient field applied at the motion frequency [96]. Preprocessing directional filtering techniques can be used to minimize shear wave interference artifacts that can affect the stiffness calculations. Mathematical algorithms are then used to calculate the mechanical properties of the tissue from the phases of the images acquired. The frequency domain equation of motion for a general, homogeneous, anisotropic, viscoelastic material relates an applied mechanical stress to the resultant strain and can be expressed as a rank-4 tensor with 21 independent complex quantities. Simplifying assumptions, like isotropy and incompressibility, allow for the calculation of mechanical properties (for example, shear modulus) to be used for clinical interpretations. Making the assumption of isotropy reduces the number of independent quantities to two; these are typically the two Lamé constants that predominantly control the longitudinal and shear strains [97]. Depending on the technique used to derive the elastograms from the original MRI images, elastograms shave typically one-third to one-fifth of the spatial resolution off the resolution of the original MRI images [97].

Due to flexibility, noninvasiveness, and potential clinical applications, the field of MRE has been rapidly evolving with new applications emerging for various organs, including the eye [97]. The elastic properties of the corneoscleral shell of an intact, enucleated, bovine globe were estimated using MRE and finite element modeling, assuming linear isotropic motion. Two-dimensional axisymmetrical model geometry was derived from a segmented two-dimensional MRI image, and estimations of Young’s modulus in both the cornea and sclera were made at various intraocular pressures, using an iterative flexural wave speed-matching algorithm. This ocular MRE study showed that it is possible to estimate the intrinsic elastic properties of the corneoscleral shell in an ex vivo bovine globe, suggesting that MRE may be useful to assess the mechanical properties of the eye and its anatomy. Further development of the technique and modeling process is needed to enhance the clinical potential of ocular MRE [96].

## Discussion

To date, most ocular MRI studies reported in the literature have been performed without the use of contrast agents, while the most successful techniques allowed for the visualization of the eye and optic nerve anatomy of animals [7,38,41,42] and humans [26-28,40,43,46] or of sites associated with infiltrated macrophages in the diseased rat eye with retinal detachment [39]. The most important imaging parameters in the unenhanced and contrast agent-enhanced ocular studies performed in human and animal studies are detailed in Appendix 1.

The smallest reported voxel sizes of the ocular MRI images acquired in vivo range between 23×23×680 μm^3^ in rat [7] and 100×200×2,000 μm^3^ in human [36] eye studies. Two rat retinal layers, an outer and an inner layer, were visualized and their perfusion was quantitatively assessed using high spatial resolution MRI techniques [7,66]. In the mouse eye, the diffusion properties within four different layers [41,42] and blood flow of two vascular layers [59] were quantitatively assessed in vivo using high spatial MRI techniques. The four retinal layers detected, from the optic nerve to the vitreous side of the wild-type mice eye, were assigned to choroid, outer nuclear layer/inner segments/outer segments, inner nuclear layer/outer plexiform layer, and nerve fiber layer/ganglion cell layer/inner plexiform layer [41]. The three mouse different blood flow retinal layers were attributed to two vascular regions corresponding to the retinal blood vessels and the choroid separated by an avascular layer corresponding to the avascular region in the retina [59]. Three retinal layers were also visualized in the human eye in vivo [31]. The signal-to-noise ratio of the images decreases with a decrease in voxel size but increases with the magnetic field strength used for MRI image acquisition. Hence, although these techniques allowed intraretinal information to be achieved in vivo, one set of rat eye MRI images, for example, required acquisition times as long as 50 min to achieve the desired high spatial resolution at the required image quality. The acquisition times of one and multiple sets of MRI images were reduced to approximately 15 min in the mice [41,59] and a few seconds in the human [31] eye studies but at the expense of reduced spatial resolution. Despite this, in the human eye study with the highest spatial resolution, signal intensity was uniform only at the back of the eye, allowing only geometric measurements, such as retinal layer thickness, to be performed on these images. To enable accurate quantitative information to be extracted from MRI images acquired using ocular diffusion, perfusion, or relaxometry protocols, which are inherently dependent on the fidelity of the measured signal intensity, techniques addressing this MRI signal non-uniformity need to be developed.

Human retinal abnormalities, including retinal detachments, have also been successfully visualized in vivo in MRI images. The pattern of such retinal detachments in the rat eye is similar to that detected in the human eye (see Figure 2B,C). Figure 4 shows different patterns of retinal detachments detected in the rat eye in vivo that were mirrored ex vivo by histology [38].

Quantitative information on the mouse [44,45] and human [46] symmetry and volumes of different ocular structures and on the retinal thickness of the human [14,31] and rat [38] eye, area of retinal detachment, and thickness of the cornea of the rat eye [38] was also obtained in vivo using high-resolution MRI techniques. Inflammatory eye disease can be detected by MRI for retinal thicknesses larger than 1,200 μm for human [14] and 200 μm for rat [38] eye studies. Retinal detachments as small as 10^5^ μm^2^ (0.1 mm^2^) were measured on MRI images of rat eyes at the early stages of EAU [38].

T_1_ [28,32,42] and T_2_ [32,42] maps of the human [28,32] and mouse [42] eye can also be extracted from ocular MRI images acquired in vivo. The mean values of the T_1_ and T_2_ relaxation times measured for different eye structures might provide quantitative information that can give insights into the mechanisms of eye diseases. Diffusion anisotropy in three different mouse retinal layers can quantitatively be monitored in vivo using MRI techniques without contrast agents [41].

Macrophages were visualized on MRI images of gel phantoms and anesthetized rat eyes at the peak of EAU. A minimum number of 12 to 286 unlabelled macrophages, each having a radius of 29 μm to 10 μm, were detected in the human macrophage gel phantom MRI studies [39]. These estimations do not take into account the possibility of colonies of macrophages affecting the signal intensity over larger distances than the vacuole itself depending on their concentration in a region and also on their susceptibility effect or on their effect on the T_1_ and T_2_ relaxation times in that region. If the cells produce changes of the physical properties of ^1^H nuclei in water molecules over distances much larger than the vacuole itself, smaller numbers of macrophages can be visualized in vivo.

MRI techniques which use contrast agents provide more quantitative information, but only Gd-DTPA-based compounds are routinely used clinically at present. Perfusion of the rat retina [65] and physiology of different rat retinal layers [66] can quantitatively be monitored in vivo using extracellular contrast agents for MRI. Visualization of sites infiltrated by macrophages in the rat retina was also possible using intracellular contrast agents for MRI [39]. MRI techniques using contrast agents were mainly applied in the rat, and further investigations are needed before their clinical usefulness is established.

Unwanted spatial variations of the signal intensity [8] can be generated if the MRI system is not correctly set up or by eye-blinking artifacts. These effects, if not taken into account, may lead to misinterpretation of pixel intensity measurements and, ultimately, to incorrect tissue identification. The SNR of MRI images is also reduced by these pixel intensity variations. These effects can be avoided and the blinking artifacts can be reduced as much as possible by setting up the MRI system and imaging protocol correctly and by careful patient positioning. The use of fast pulse sequences [26], optimization of the image reconstruction schemes [98], and careful positioning of the human subject in the scanner [26,27,33] were proposed to reduce eye-blinking artifacts and to improve the SNR of clinical ocular MRI images.

The accuracy of geometric measurements is limited by the spatial resolution of MRI images, which is dependent primarily on the SNR and hence acquisition time when multiple signal averaging is used to improve the SNR [8]. This may well represent the limiting factor for clinical MRI studies in ophthalmology.

Higher magnetic field strengths improve the SNR of MRI images, allowing images with higher spatial and/or temporal resolution to be acquired. However, the tissues imaged are exposed to greater specific absorption rates in higher magnetic field strengths, since the specific absorption rate scales approximately with the square of the static magnetic field. In a study performed on over 500 human subjects, it was shown that measured vital sign changes related to a sitting or supine position are larger than those associated with exposure to a static magnetic field of up to 8 T [99]. Increase in the magnetic field strength dramatically increases magnetic susceptibility effects and related artifacts, such as errors in the correct attribution of tissue location, shape distortion, and signal intensity loss within the tissues. To minimize these effects and to avoid artifacts, MRI systems need to be carefully set up and the imaging protocols have to be tested on adequate phantoms before clinical MRI. Good shimming of the magnetic field together with the appropriate selection of the imaging protocol, the acquisition parameters, and the imaged geometry are, consequently, important in high-field clinical MRI. Other drawbacks of high-field MRI include acoustic noise and dielectric resonance [100]. These problems have largely been resolved by improvements in hardware and software in MRI scanners [100]. Higher magnetic field strengths also affect the relaxation properties and chemical shift in the region imaged [100]. At higher field strengths, T_1_ contrast tends to be minimized and T_2_ contrast tends to be emphasized, while the chemical shift separation is more pronounced [100].

Another possibility of increasing the SNR of MRI images is the special design of the transmit/receive RF coils. The design and use of cryo-cooled eye-shaped coils or multichannel phased-array coils would allow further increase in the SNR of MRI images compared to that obtained at the moment clinically or in animal research. In conclusion, high spatial and temporal resolution MRI techniques with and without contrast agents provide qualitative and quantitative information with clinical potential in ophthalmology.

## Appendix 1. Details of the most relevant ocular MRI studies.

To access the data, click or select the words “Appendix 1.” This will initiate the download of a compressed (pdf) archive that contains the file.
